# Supplementary material for: Extraversion Is Associated With Lower Brain Beta-Amyloid Deposition in Cognitively Normal Older Adults
Source: Front Aging Neurosci. 2022 Jul 13;14:900581. doi: 10.3389/fnagi.2022.900581 (PMC9325961; doi:10.3389/fnagi.2022.900581)
Supplement: Supplementary file 1 [file Data_Sheet_1.docx]

Supplementary Material

# Supplementary Tables

**Supplementary Table 1. Personality scores for five factor personality domains.**

_________________________________________________________________________

**Personality N Minimum Maximum Mean Std. Deviation**

_________________________________________________________________________

**All subjects**

extraversion 90 1.700 4.700 3.30333 .766057

agreeableness 90 2.700 7.000 4.13877 .632177

conscientiousness 90 2.000 5.000 3.88611 .656882

openness 90 1.444 4.800 3.76694 .658644

neuroticism 90 1.000 4.700 2.63086 .896018

**Young subjects**

extraversion 33 1.700 4.700 3.39697 .806414

agreeableness 33 2.700 5.000 4.10640 .593685

conscientiousness 33 2.200 5.000 3.90909 .655441

openness 33 1.444 4.700 3.81515 .824279

neuroticism 33 1.300 4.700 2.90370 1.036344

**Older subjects**

extraversion 57 1.800 4.500 3.24912 .743573

agreeableness 57 2.800 7.000 4.15750 .657856

conscientiousness 57 2.000 5.000 3.87281 .663165

openness 57 2.000 4.800 3.73904 .546846

neuroticism 57 1.000 4.500 2.47290 .770047

_________________________________________________________________________

# Supplementary Figures


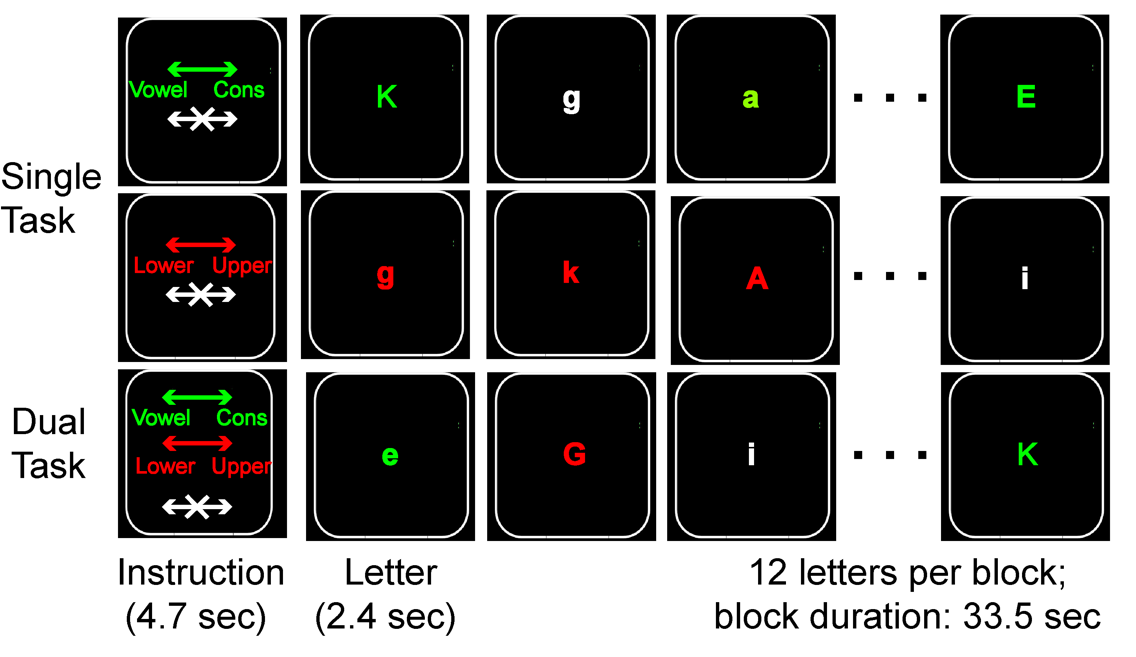


**Supplementary Figure 1.** **Schematic diagram of task-switching fMRI task**
